# Voluntary wheel running during adolescence prevents the increase in ethanol intake induced by social defeat in male mice

**DOI:** 10.1007/s00213-023-06461-0

**Published:** 2023-09-22

**Authors:** Marina D. Reguilón, Carmen Ferrer-Pérez, Carmen Manzanedo, José Miñarro, Marta Rodríguez-Arias

**Affiliations:** 1 Unit of Research on Psychobiology of Drug Dependence, Department of Psychobiology, Faculty of Psychology, Universitat de València, Avda. Blasco Ibáñez 21, 46010 Valencia, Spain; 2 Departmento de Psicología Evolutiva, Facultad de Psicología, Universitat de València, Valencia, Spain

**Keywords:** Physical exercise, Wheel running, Social stress, Resilience, Ethanol, BDNF, Self-administration

## Abstract

**Rationale:**

Exposure to social defeat (SD) induces a depressive phenotype, increased ethanol seeking and consumption, accompanied by activation of the neuroinflammatory response. However, a resilient response can be potentiated through physical exercise in the form of voluntary wheel running (VWR) during or after exposure to social stress. Therefore, the aim of this study was to test whether physical exercise during adolescence prior to being exposed to SD can enhance resilience to the increase in ethanol intake.

**Methods:**

Male mice had access to VWR during adolescence and the effects of social defeat (4 sessions every 72 h) on oral ethanol self-administration (SA) was evaluated. Based on the social interaction test, mice were classified as resilient or susceptible to depressive-like behavior. Two weeks after the last encounter, mice were subjected to the drinking in the dark and oral ethanol SA paradigms. Mice were then sacrificed to measure brain-derived neurotrophic factor (BDNF) levels in the striatum and hippocampus.

**Results:**

As expected, susceptible mice increased ethanol intake in the oral SA protocol. However, susceptible mice in the exercise condition did not increase ethanol intake, showing similar consumption and motivation for ethanol than the control and resilient groups. On the other hand, decreased BDNF levels were observed in susceptible mice in both experimental conditions compared to the control groups after ethanol SA.

**Conclusions:**

The pre-exposure of VWR prevented the increase in consumption and motivation for ethanol induced by SD in susceptible mice. On the other hand, it appears that VWR did not exhibit any significant long-term effects on BDNF signaling, which is mainly affected in susceptible mice after ethanol intake.

**Supplementary Information:**

The online version contains supplementary material available at 10.1007/s00213-023-06461-0.

## Introduction

For decades, socially stressful experiences have been associated with the deterioration of mental health. A clear example is how social stress induces neuronal and behavioral alterations that increase vulnerability in the addictive process (Montagud-Romero et al. 2018; Newman et al. 2018; Nikulina et al. 2014; Vasconcelos et al. 2015). The resident-intruder paradigm, also known as social defeat, is a preclinical model validated for rodents and based on social hierarchy and dominance (Koolhaas et al. 2013; Miczek 1979). Long-term defeat is associated with cognitive impairment, social deficits, anxiety, anhedonia, depressive-like behavior, and also increased vulnerability to drug use (Bath et al. 2017; Montagud‐Romero et al. 2018; Stein et al. 2017). Exposure to social defeat (SD) in rodents causes a short and long increase and escalation of ethanol consumption (Norman et al. 2015; Reguilón et al. 2020, 2021a, b; van Erp and Miczek 2001). These changes in ethanol intake have been associated with social stress-induced neuroadaptations in hypothalamic, extrahypothalamic, and mesocorticolimbic circuits related to stress and reward (Holly et al. 2015; Hwa et al. 2016; Laine et al. 2017; Newman et al. 2018).

However, as in humans, not all rodents develop depressive-like behaviors or increased drug seeking and taking induced by SD. Resilience is the process and outcome of successfully adapting to difficult or challenging life experiences, especially through the flexibility of our body's neural circuitry and biological responses (for review see Bath et al. 2017; Dantzer et al. 2018; Han and Nestler 2017). Resilient mice appear to have a different coping strategy than their more susceptible conspecifics. The characterization of phenotypes resilient/susceptible to social stress could serve as a means to devise strategies aimed at preventing the onset of problems such as anxiety and depression, and reducing the likelihood of developing vulnerability to drug abuse. A resilient response has been associated with reduced reactivity of the hypothalamic–pituitary–adrenal (HPA) axis to chronic stress, with positive neuroadaptations in structures that form the limbic system, increased neurogenesis in the hippocampus, and a different immune response to susceptible mice (Ballestín et al. 2021; McEwen 2016; Nasca et al. 2019; Reguilón et al. 2021b). We have previously shown that resilient mice to depressive-like behaviors did not show an increased in the conditioned and operant response to the rewarding effects of cocaine nor an increase in SD-induced ethanol intake as susceptible mice did present (Ballestín et al. 2021; Giménez-Gómez et al. 2021; Reguilón et al. 2021b). We have also observed that resilient mice showed a reduced neuroinflammatory response, with decreases in IL-6 levels and increases in CX3CL1 levels in the prefrontal cortex, striatum and hippocampus (Ballestín et al. 2021; Giménez-Gómez et al. 2021; Reguilón et al. 2021b).

The World Health Organization (WHO 2020) report on physical activity shows that it reduces symptoms of depression and anxiety, and improves thinking, learning and judgment skills. In addition, the WHO emphasizes physical exercise at all stages of the life cycle, showing that in adolescence, physical activity improves cognitive outcomes (academic performance and executive function) and mental health (reduction of depressive symptoms). In preclinical studies, voluntary exercise in wheels has been shown to have a potentiating effect on learning and neurogenesis, resulting in increased neurotrophic factors and molecular signaling changes, as well as a reduction in depressive-like behaviors (Calpe-López et al. 2022; Mul 2018; Salam et al. 2009). Some studies have shown that physical exercise regulates the HPA axis (Lynch et al. 2019). Prolonged exposure to physical exercise generated an adaptive response that decreased anxiogenic-type responses (Pietrelli et al. 2018a). In addition, voluntary wheel running (VWR) had beneficial effects that prevented restraint stress-induced anxiety/depression behaviors and memory impairment (Lapmanee et al. 2017). Also, it has been observed that physical exercise during adolescence reduced serum corticosterone levels in the hippocampus induced by maternal separation in adult rats (Zolfaghari et al. 2021). Other studies focusing on the reward system have shown that physical exercise alters the transcription of genes in the mesolimbic reward pathway that induce changes in the rewarding properties of drugs of abuse and facilitate successful stress management (Greenwood et al. 2011). In relation to the rewarding effects of ethanol, physical exercise has been observed to significantly reduce ethanol consumption and preference in paradigms such as two-bottle choice (TBC), operant oral self-administration (SA) and conditioned place preference (CPP; Darlington et al. 2014, 2016; Ehringer et al. 2009; Gallego et al. 2015; Reguilón et al. 2020).

Brain-derived neurotrophic factor (BDNF) is considered critical for neuronal and synaptic plasticity throughout the nervous system (Baj et al. 2012; Erickson et al. 2012; Liu and Nusslock 2018; Vaynman et al. 2004). BDNF has been shown to be central to the development of addictive behavior and SD-induced depressive-like behaviors, as it is involved in reward circuitry, specifically in ventral tegmental area (VTA) dopaminergic neurons projecting into the nucleus accumbens (NAc; for review see Koo et al. 2019; Krishnan 2014; Nikulina et al. 2014). Physical activity in different modalities (with or without load and over short and long distances) has great benefits on cognitive functions involving the hippocampus and BDNF signaling in the hippocampus (Ferrer-Pérez et al. 2022; Lee et al. 2012). Physical exercise increases cortical, hippocampal and striatal BDNF expression in rodents subjected to physical and social stress, and at different periods of life, resulting in increased neuroplasticity and prevention of neuronal death (Ferrer-Pérez et al. 2022; Lee et al. 2012; Marais et al. 2009; Pietrelli et al. 2018b; Sasse et al. 2013).

We have previously reported that voluntary exercise during and after exposure to SD effectively reduced ethanol consumption and neuroinflammation associated with SD exposure in adult mice (Reguilón et al. 2020). In this line, a recent report confirmed the role of physical exercise in buffering the increase in the conditioned rewarding effects of cocaine induced by SD (Ferrer-Pérez et al. 2022). To date, there are no studies to evaluate the protective role of physical exercise during adolescence prior to exposure to SD in enhancing a resilience response. Potentially protective interventions during adolescence affect brain development and shape neural circuits that regulate later stress responses. Providing tools to counteract the adverse effects of SD is essential to reduce vulnerability to mental disorders such as depression or substance use disorder (El Rawas et al. 2020). Therefore, the aim of this study was to evaluate the role of physical exercise during adolescence as a preventive tool against the development of vulnerability to the rewarding and motivational effects of ethanol induced by social stress in stress-susceptible rodents. In addition, BDNF expression in the hippocampus and striatum was evaluated, since physical exercise has been related to an increase in the expression of this neurotrophic factor that may influence these structures and modify the associative learning cues involved in the oral SA of ethanol and in the neuroplasticity associated with the learning of drug use (Baruch et al. 2004; Eisenstein and Holmes 2007; Greenwood et al. 2011).

## Methodology

### Animals

A total number of 74 adult male C57BL/6 mice (Charles River, France) were delivered to our laboratory at postnatal day (PND) 21. Experimental mice were housed in groups of five in plastic cages (27 × 27 × 14 cm) during the entire experimental procedure. OF1 adult mice (Charles River, France) were used as aggressive opponents (*n* = 20) and were individually housed in plastic cages (21 × 32 × 20 cm) for at least one month prior to initiation of the experiments in order to heighten aggression (Rodríguez-Arias et al. 1998). All mice were housed in controlled laboratory conditions: constant temperature and humidity and a reversed light schedule (white light from 8:00 to 20:00). Food and water were available ad libitum to all the mice used in this study, except during behavioral tests. All procedures were conducted in compliance with the guidelines of the European Council Directive 2010/63/UE regulating animal research and were approved by the local ethics committees of the University of Valencia (number 2017-VSC-PEA-00224, on December, 11th 2017).

### Drugs

For the drinking in the dark and oral SA procedures, absolute ethanol (Merck, Madrid, Spain) was diluted in water using a 20% (v/v) ethanol solution.

### Experimental design

In the experimental design, all the mice were delivered to our laboratory PND 21 and were housed in regular condition throughout the study. Four experimental groups were randomly assigned, on the one hand, a control group and a defeated group (which remained undisturbed until the PND 47), and on the other hand, a control group and a defeated group which were exposed VWR 1 h of exercise for three days a week (Monday, Wednesday, and Friday) from PND 26 to PND 46. Subsequently, all mice were exposed to the SD procedure or exploration from PND 47 to 56. 24 h after the last SD episode, mice performed the social interaction test (SIT) to evaluate depressive-like behaviors and were characterized as resilient or susceptible depending on their social behavior. From this point on, the SD groups are divided into susceptible and resilient groups, forming six experimental groups: CTRL; SD-R; SD-S; VWR-CTRL; VWR-SD-R; VWR-SD-S. Three weeks after the last defeat, the mice initiated the drinking in the dark (DID) test for four days and, in the following week, mice initiated the ethanol SA protocol for approximately 22 days. At the end of this test, all the mice were sacrificed to obtain the hippocampus and striatum for further analysis of the BDNF levels.

**Fig. 1:**
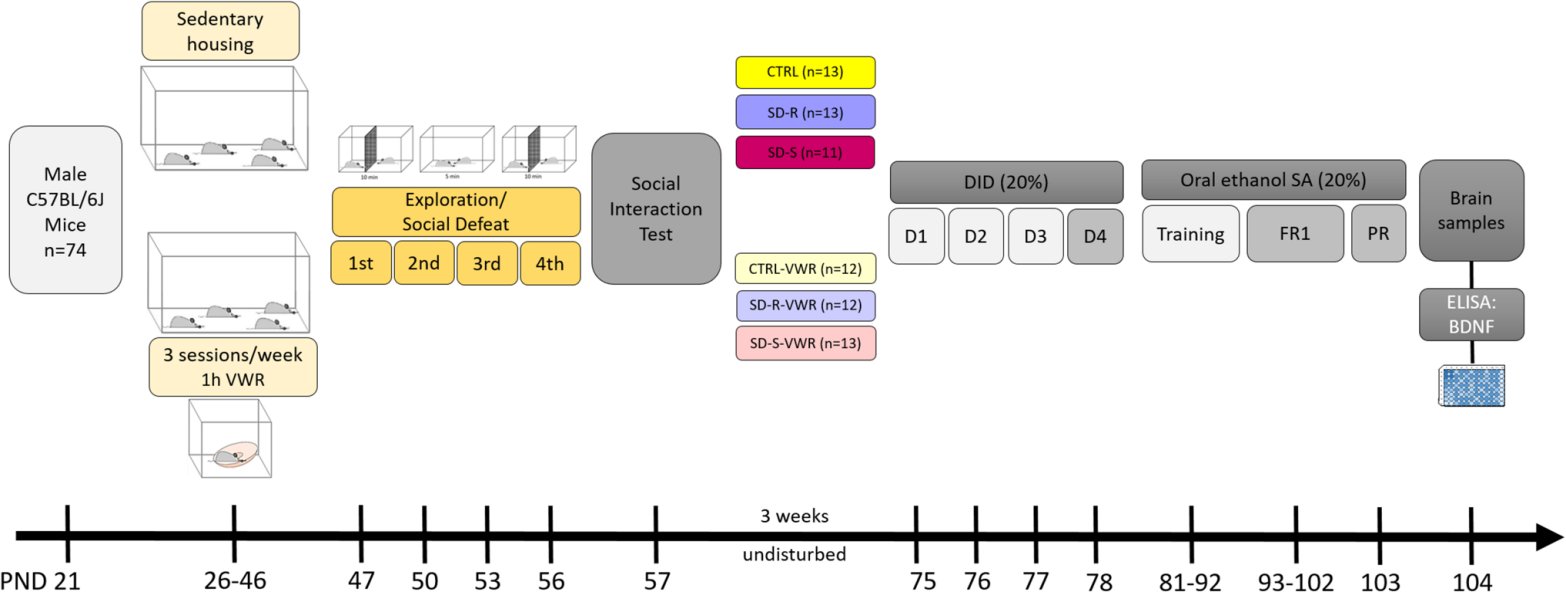
Experimental design

The experimental design is depicted in Fig. 1.

### Procedure and apparatus

#### Low-profile running wheel

The type of wheel used was the low-profile running wheel (Med Associates Inc.), which rotates on a central axis in a horizontal plane, allowing physical activity to be carried out through natural exercise as in spontaneous locomotion. We used 8 low-profile running wheels, each of which was placed individually within a plastic cage that was different from the mice’s house cage. In order to avoid competition for the running wheel, all animals in the wheel condition (VWR + CTRL and VWR + SD) were distributed in batches of 8 for one hour per day, three times a week (Monday, Wednesday, and Friday). These wheels have an ideal size (10.25 × 15.5 × 13.7 cm) to be introduced into plastic cage measuring 21 × 32 × 20 cm and are linked to a monitoring system (Hub) that runs on batteries and can register the activity through a set of programs (Wheel Manager Software).

#### Procedure of social defeat (SD)

During early adulthood, mice in the stress/defeat groups were exposed to four episodes of SD at PND 47, 50, 53 and 56. At each PND, mice were exposed to SD for 25 min. The SD consisted of three phases, the initial phase began by introducing the “intruder” (the experimental animal) into the home cage of the “resident” (the aggressive opponent) for 10 min (Tornatzky and Miczek 1993). During this initial phase, the intruder was protected from attack, but the wire mesh walls of the cage allowed for social interactions and species-typical threats from the male aggressive resident, thus facilitating instigation and provocation (Covington and Miczek 2001). In the second phase, the wire mesh was removed from the cage to allow confrontation between the two mice over a 5-min period. The second phase of each SD protocol was video-recorded and ethologically analysed. Finally, the wire mesh was returned to the cage to separate the two mice once again for another 10 min to allow for social threats by the resident. The non-stressed exploration groups underwent the same protocol, but without the presence of a “resident” mouse in a clean cage. The intruder mice were exposed to a different aggressor mouse during each SD episode. The criterion used to define an animal as defeated was the adoption of a specific posture signifying defeat, characterized by an upright submissive position, limp forepaws, upwardly angled head, and retracted ears (Miczek et al. 1982; Rodríguez-Arias et al. 1998). A detailed description of these behaviors can be found in Rodríguez-Arias et al. (1998).

#### Social interaction test (SIT)

The SIT is based on the social approach-avoidance test previously described by Berton et al. (2006) with temporal modifications performed by Henriques-Alves and Queiroz (2015). The test took place 24 h after the last SD during dark light cycle and in a different environment of the confrontation sessions. First, mice were transferred to a quiet, dimly lit room 1 h before the test was initiated. After habituation, each animal was placed in the center of a square arena (white Plexiglas open field, 30 cm on each side and 35 cm high) and its behavior was monitored by video (EthoVision XT 11, 50 fps; camera placed above the arena). Mice were allowed to explore the arena twice, for 600 s in each session, during two different experimental sessions. In the first (object session), an empty perforated Plexiglas cage (10 × 6.5 × 35 cm) was placed in the middle of one wall of the arena. In the second session (social session), an unfamiliar C57BL/6 male mouse was introduced into the cage as a social stimulus. Before each session, the arena was cleaned with 5% alcohol solution to minimize odor cues. Between sessions, the experimental mouse was removed from the arena and returned to its home cage for 2 min.

Arena occupancy during object and social sessions were determined using the animals’ horizontal positions, determined by commercial video tracking software (EthoVision XT 11, Noldus). Conventional measures of arena occupancy, such as time spent in the interaction zone and corners, were quantified. The former is commonly used as social preference-avoidance score and is calculated by measuring the time spent in a 6.5 cm wide corridor surrounding the restraining cage. Corners were defined as two squares of similar areas on the opposite wall of the arena.

#### Drinking in the dark (DID)

Following the basic paradigm of Rhodes et al. (2005), the test consists of two phases. The first is habituation, in which the mice are removed from their cages to be housed individually 2 h per day for one week to habituate them to the cages and to the sipper tubes (containing a ball bearing at the end to prevent leakage and to be used throughout the test) with water. In the second phase of the protocol, the test begins three hours after lights out. The mice are then housed in the individual cages and exposed to the 10 ml graduated tubes containing a 20% (v/v) ethanol solution. This procedure lasts two hours. After this two-hour period, the mice are returned back to their grouped cages, with food and water bottles ad libitum. This procedure is repeated on days 2 and 3, and on day 4, the procedure lasts for 4 h. In addition, immediately after each day, the volume consumed was recorded. A fresh ethanol solution is prepared each day. In our case, we will maintain the protocol for two consecutive weeks, one for habituation with water and one for ethanol testing.

#### Oral ethanol self-administration (SA)

This procedure is based on that employed by Navarrete et al. (2014). Oral ethanol SA was carried out in 8 modular operant chambers (MED Associated Inc., Georgia, VT, USA). Software package (Cibertec, SA, Spain) controlled stimulus and fluid delivery and recorded operant responses. The chambers were placed inside noise isolation boxes equipped with a chamber light, two nose-poke holes, one receptacle to drop a liquid solution, one syringe pump, one stimulus light and one buzzer. Active nose-pokes delivered 20 μl of fluid combined with a 0.5 s stimulus light and a 0.5 s buzzer beep, which was followed by a 6 s time-out period. Inactive nose-pokes did not produce any consequence.

This protocol did not involve daily water and food deprivation. The restriction of food and water was solely enforced during the sessions within the operant chambers (1 h or 2 h depending on the experimental phase). All sessions included access to both active and inactive nose-pokes.

To evaluate the consequences of SD on the acquisition of oral ethanol SA, mice underwent an experiment carried out in three phases: training, fixed ratio 1 (FR1) and progressive ratio (PR) with a 20% ethanol concentration.

##### Training phase (12 days)

Mice were trained to discriminate between both nose-pokes and to select the active nose-poke to receive a 20 μl of 20% (v/v) ethanol reinforcement. During this phase, the mice were exposed to the operant chamber for approximately 12 consecutive days for 1 h per day, with each session being repeated every 24 h. In order to assess the learning of the mice and determine their acquisition of operant behavior, several requirements must be achieved, including demonstrating a preference for the active nose-poke by a minimum of 60% over the inactive nose-poke for at least 3 consecutive days and exhibiting a deviation of less than 30 points during the last 3 days of this phase. The consumption of liquid is not taken into consideration during this session.

##### FR1 (10 days)

This phase lasted for 10 consecutive days and aimed to evaluate the quantity of responses in the active nose-poke and the intake of 20% (v/v) ethanol. The number of effective responses and ethanol consumption (μl) were measured for each session. The remaining ethanol in the receptacle was collected and measured using a micropipette to account for each mouse's voluntary liquid consumption in each session.

##### PR (1 day)

In the third and final phase, a PR session was conducted to establish the breaking point (BP) for each animal (the maximum number of nose-pokes each animal is able to perform in order to earn one reinforcement). This phase of the procedure was conducted on a single day and lasted for 2 h. The response requirement to achieve reinforcements escalated according to the following series: 1-2-3-5-12-18-27-40-60-90-135-200-300-450-675-1000. To evaluate motivation toward ethanol consumption, the breaking point was calculated for each animal as the maximum number of consecutive responses it performed to achieve one reinforcement according to the previous scale (Navarrete et al. 2014). For example, if an animal activated the nose-poke a total of 108 times, this meant that it was able to respond a maximum of 40 times consecutively for one reinforcement. Therefore, the BP value for this animal would be 40.

#### Immunoassay analysis (ELISA)

Samples from the hippocampus and striatum were obtained 24 h after SA. To obtain tissue samples, mice were sacrificed by cervical dislocation and then decapitated. Brains were rapidly removed and the hippocampus and striatum dissected with a brain slicer matrix with 1 mm coronal section slice intervals using mouse brain atlas coordinates (Franklin and Paxinos 2008; Heffner et al. 1980), which were then kept in dry ice until storage at -80ºC. To dissect the striatum, coronal slice sections approximating stereotaxic coordinates between 1.42 and 0.14 mm were selected from Bregma, and to dissect the hippocampus, approximate stereotaxic coordinates between -1.22 and -3.40 mm from Bregma were followed. Before BDNF determination, brains were homogenized and prepared following the procedure described by Alfonso-Loeches et al. (2010). Frozen brain cortices were homogenized in 250 mg of tissue/0.5 ml of cold lysis buffer (1% NP-40, 20 mM Tris–HCl pH 8, 130 mM NaCl, 10 mM NaF, 10 μg/ml aprotinin, 10 μg/ml leupeptin, 40 mM DTT, 1 mM Na3VO4, and 10 mM PMSF). Brain homogenates were kept on ice for 30 min and centrifuged at the maximum speed for 15 min; the supernatant was collected, and protein levels were determined by the Bradford assay from ThermoFisher (Ref: 23227).

The concentrations of BDNF in homogenized extracts were measured with commercial enzyme-linked immunosorbent assay (ELISA) kits in 96-well strip plates (Biosensis, 211BEK-2211-1P). We determined BDNF concentration in the hippocampus and striatum. All reagents and standard dilutions were prepared following the manufacturer’s instructions. To determine absorbance, we employed an iMark microplate reader (Bio-RAD) controlled by Microplate Manager 6.2 software. Optical density of plates was read at 450 nm and the final results were calculated using a standard curve following the manufacturer's instructions. Total protein concentrations were determined using the Pierce BCA Protein Assay Kit (ThermoFisher Scientific) to determine the number of picograms of BDNF. Data are expressed as pg/mg of protein for tissue samples.

### Statistical analysis

Mice were previously classified into resilient and susceptible groups based on the SIT. A ratio was calculated by considering the time spent by an experimental mouse in the interaction zone when a social target is present divided by the time it spends in the interaction zone when the target is absent. A ratio equal to 1 means that equal time has been spent in the presence versus absence of a social target. Based on the regular behavior of control C57BL/6 mice, animals with a ratio under 1 are classified as susceptible, while those with a ratio equal to or higher than 1 are classified as resilient (Golden et al. 2011). In order to analyse the proportions of social interaction, a UNIANOVA with two between-subjects variable—SD, with three levels (CTRL, SD-S and SD-R) and VWR with two levels (not exposed to VWR and exposed to VWR). We also used a three-way ANOVA with two between-subjects variables –SD with three levels (CTRL, SD-S and SD-R) and VWR with two levels (not exposed to VWR and exposed to VWR)– and a within-subjects variable –Session, with two levels of SI session – for analysis of total time in the interaction zone when the target is absent or present for each session. Moreover, a K-means cluster analysis was conducted to determine the distribution of animals within each condition, where they were sedentary or in VWR condition, based on their higher or lower average ethanol intake during FR1 schedule of SA.

The data from the ethological analyses of resident and intruder mice were analysed by a three-way ANOVA with two between-subjects variables –SD with two levels (SD-S and SD-R) and VWR with two levels (not exposed to VWR and exposed to VWR)– and a within-subjects variable –Days with two levels: 1^st^ and 4^th^ agonistic encounter–.

To analyse acquisition of ethanol SA, a three-way ANOVA was performed with two between-subjects variables –SD with three levels (CTRL, SD-S and SD-R) and VWR with two levels (not exposed to VWR and exposed to VWR)– and a within-subjects variable –Days, with ten levels of FR1–. The effects of SD and VWR on BP values and ethanol consumption during PR was analysed by a UNIANOVA, with two between-subjects variables –Stress and VWR.

The data of the BDNF levels were analysed using a UNIANOVA with two between-subjects variables –Stress, with three levels (Control, Resilient and Susceptible;) and VWR, with two levels (not exposed to VWR and exposed to VWR).

Pearson’s coefficient was calculated to determine correlation between the ethanol consumption variable (during SA FR1 schedule) and BDNF levels in the brain areas studied.

In all the studies, following the ANOVA, Bonferroni post-hoc tests were calculated whenever required. All statistical analyses were performed using SPSS Statistics v.26 and graph design with GraphPad Prism (v8; GraphPad Software Inc., CA, USA). Data were expressed as mean ± SEM and a value of *p* < 0.05 was considered statistically significant.

## Results

### Classification between susceptible and resilient mice based on their social interaction ratio

**Fig. 2:**
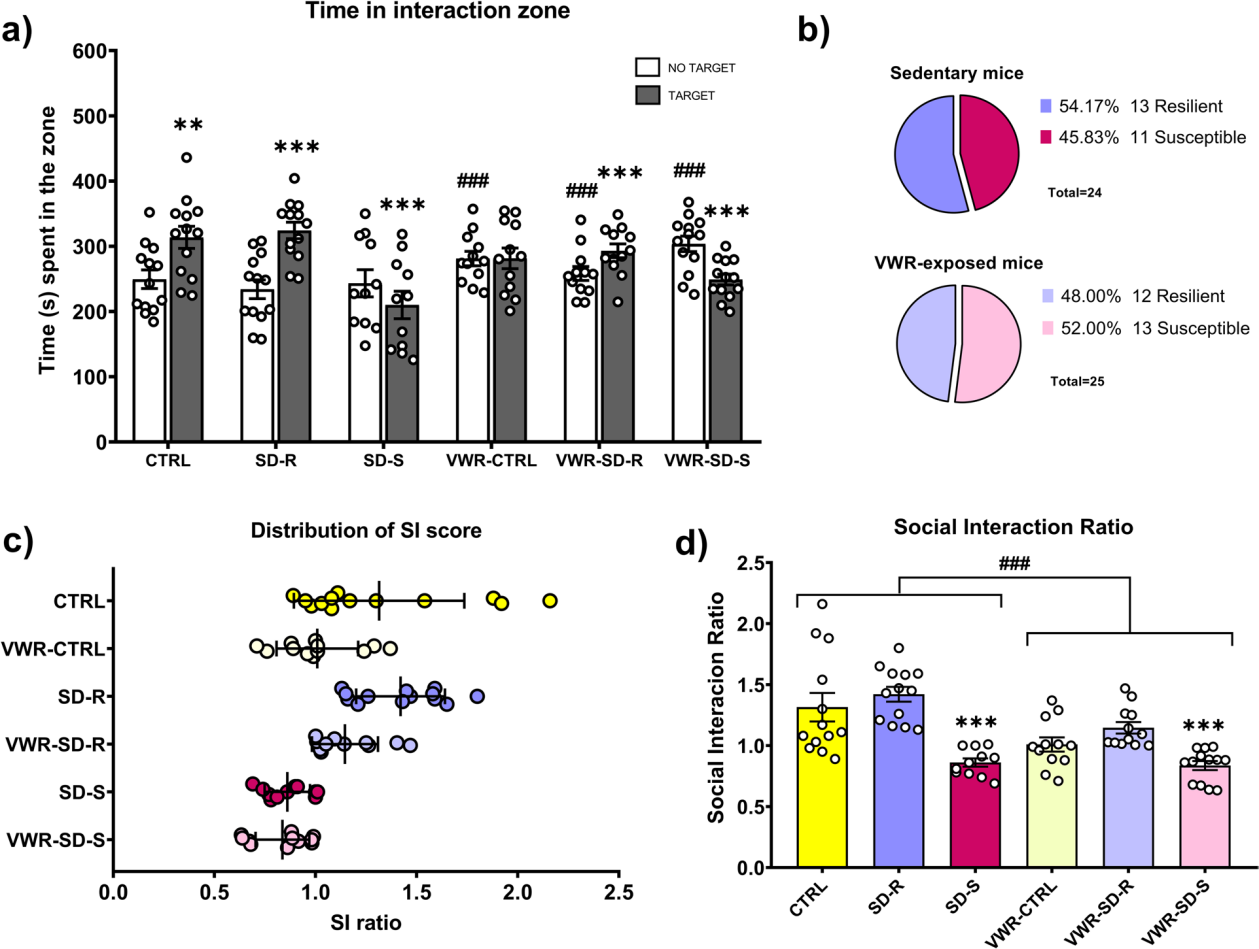
Social defeat stress induces avoidance behavior in susceptible mice. **a** Time between no target and target sessions in the interaction zone. **b** Percentages of resilient and susceptible mice in both conditions (sedentarism and VWR-exposure). **c** Distribution of social interaction scores. **d** Repeated social defeat stress results in a spectrum of avoidance behavior, divided between susceptible and resilient phenotypes based on their social interaction ratio score. Error bars represent means ± SEM. ***p < 0.001, **p < 0.05 represent significant differences between no target vs. target session. ###*p* < 0.001 represent significant differences between sedentary vs VWR-exposed mice

The analysis of variance (ANOVA) conducted for the total duration in the interaction zone, regardless of whether the target is present or absent, for each session showed an effect of the interactions Session × SD [F(2,68) = 26.586; *p* < 0.001] and Session × VWR [F(1,68) = 14.830; *p* < 0.001] (Fig. 2a). The post-hoc comparison revealed that both the control and resilient mice spent a greater amount of time in the social interaction zone during the social session as compared to the object session (*p* < 0.01 and *p* < 0.001, respectively). In contrast, susceptible mice spent less time in the social interaction zone during the social session (*p* < 0.001). Furthermore, when the social object was present, control and resilient mice spent more time in the social interaction zone than susceptible mice.

In addition, during the object session, mice exposed to VWR spent more time in the social interaction zone compared to sedentary mice (*p* < 0.001). Accordingly, sedentary mice spent more time in the social interaction zone during the object session compared to the social session (*p* < 0.001).

The ANOVA of the social interaction ratio performed 24 h after the last defeat showed an effect of the variables SD [F(2,68) = 21.888; *p* < 0.001] and VWR [F(1,68) = 13.463; *p* < 0.001] (Fig. 2d). The post-hoc comparison revealed a higher score for social interaction among control mice (CTRL and VWR-CTRL groups) and resilient mice (SD-R and VWR-SD-R groups) in comparison with susceptible mice (SD-S and VWR-SD-S groups; *p* < 0.001 in all cases). Moreover, all sedentary mice (CTRL, SD-R and SD-R groups) showed a greater social interaction score in comparison with all mice exposed to physical exercise (VWR-CTRL, VWR-SD-R and VWR-SD-S groups; *p* < 0.001). The distribution of the mice in accordance with to the SIT can be observed in Fig. 2.c.

Following the SIT calculation criteria (Fig. 2b), the CTRL group (*n* = 13) showed a mean ratio higher than 1. In the SD group of mice (*n* = 24), 45.83% of the mice showed a ratio under 1, which classifies them as susceptible (SD-S) mice (*n* = 11), and the remaining 54.17% of the mice showed a ratio equal to or higher than 1, which classifies them as resilient (SD-R) mice (*n* = 13). Moreover, the VWR-CTRL group (*n* = 12) showed a mean ratio higher than 1. In the VWR-SD group of mice (*n* = 25), 53.85% of the mice showed a ratio under 1, which classifies them as susceptible (VWR-SD-S) mice (*n* = 13), and the remaining 46.15% of the mice showed a ratio equal to or higher than 1, which classifies them as resilient (VWR-SD-R) mice (*n* = 12).

### Exposure to physical exercise does not affect behavior during SD

For the time employed in Avoidance/Flee behaviors by resilient or susceptible intruder mice, the ANOVA showed an effect of the variable Days [F (1,45) = 208.200; *p* < 0.001]. All intruder mice increased the time spent in Avoidance/Flee behavior during the 4^th^ SD (*p* < 0.001). The ANOVA conducted for the Defensive/Submissive behaviors showed an effect of the interaction Days × SD × VWR [F (1,45) = 4.950; *p* < 0.05]. VWR-exposed susceptible mice spent less time in Defensive/Submissive behavior during the 1^st^ SD compared to resilient mice also exposed to VWR (*p* < 0.05). Moreover, the post-hoc comparisons revealed an increase in time spent on this behavior during the 4^th^ SD in the SD-R (*p* < 0.001), SD-S (*p* < 0.01) and VWR-SD-S (*p* < 0.001) groups with respect to the 1^st^ social defeat.

For the time employed in Attack behavior by resident mice, the ANOVA showed an effect of the variable Days [F (1,45) = 88.002; *p* < 0.001]. All resident mice that were present during the 4^th^ SD (*p* < 0.001) showed an increase in the duration of their attack behavior. The ANOVA for the Threat behavior showed an effect of the interaction Days × Stress × VWR [F (1,45) = 5.837; *p* < 0.05]. Resident mice were less threatening to VWR-exposed resilient mice during the 4^th^ SD compared to sedentary resilient mice (*p* < 0.05).

All of these analyses are shown in Table 1.

**Table 1:**
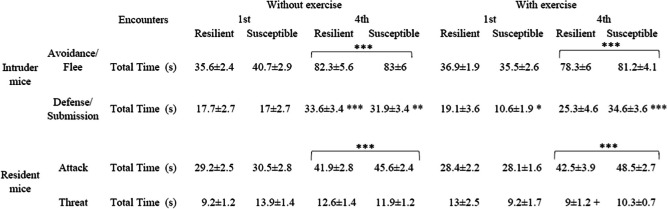
Ethological analyses of social defeat. Behavior of intruder and resident mice during 5-min agonistic encounters

***p < 0.001, **p < 0.01 significant differences between the 1^st^ and 4^th^ SDs. + *p* < 0.05 significant differences between the sedentary and VWR-exposed groups

### Voluntary wheel running during adolescence decreased ethanol consumption in the DID paradigm

**Fig. 3:**
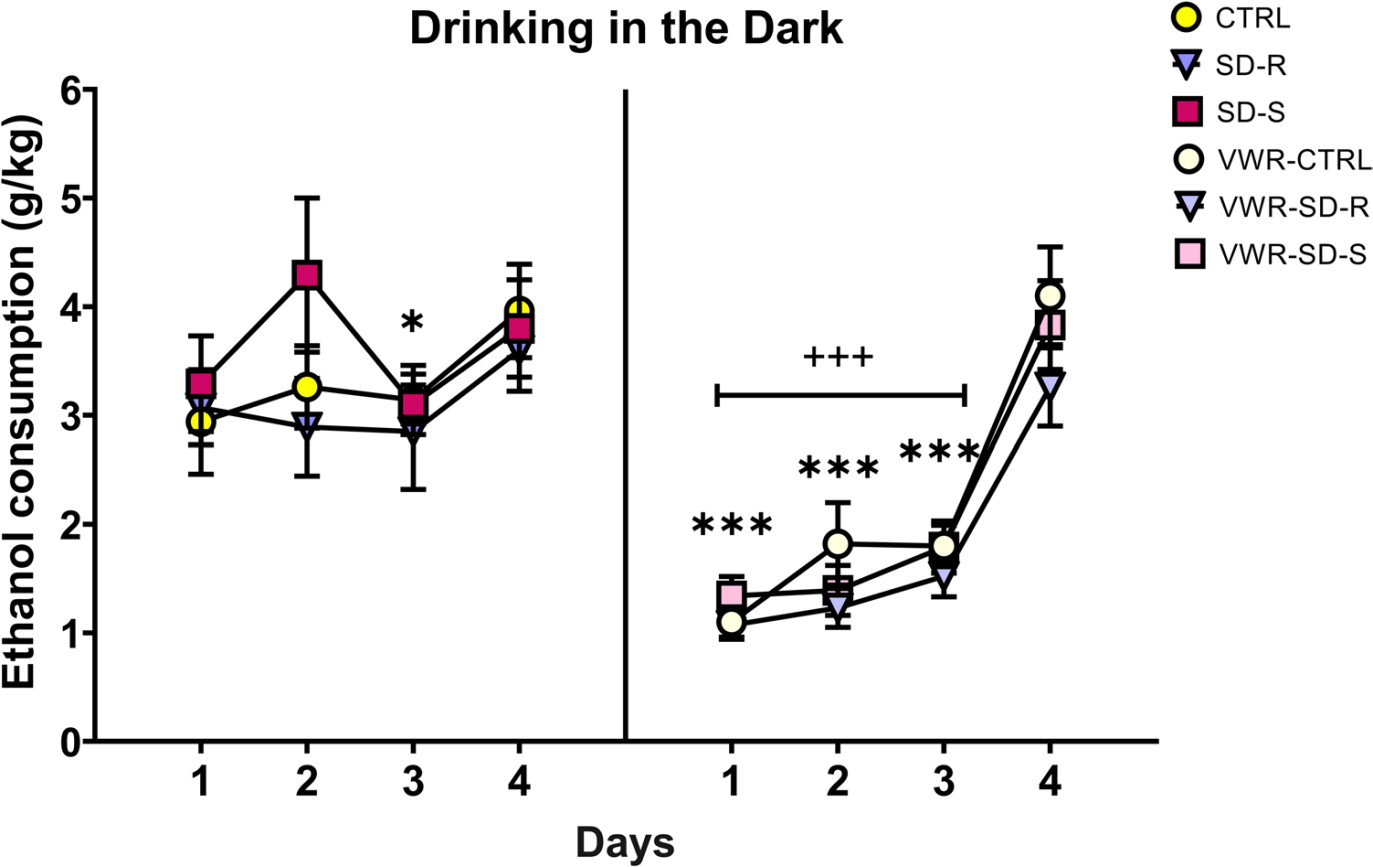
Effect of adolescent VWR on ethanol intake during DID. The dots represent means and the vertical lines ± SEM of the g/kg of ethanol at 20% consumed. *** *p* < 0.001, *p < 0.05 significant difference with Day 4; +  +  + *p* < 0.001 significant difference between VWR groups vs. non-VWR groups

The ANOVA for ethanol consumption during the DID paradigm revealed a significant effect of the interaction Days × VWR [F(3,204) = 11.664; *p* < 0.001] (Fig. 3). The post-hoc comparison showed that sedentary mice consumed significantly more ethanol during Day 4 compared to Day 3 (*p* < 0.05). Equally, VWR-exposed mice consumed significantly more ethanol on day 4 compared to Days 1, 2 and 3 (p’s < 0.001). However, sedentary mice consumed more ethanol during Days 1, 2 and 3 compared to mice trained in VWR (p’s < 0.001).

### Susceptible mice that performed VWR showed greater resistance to the increased consumption and motivation for ethanol induced by social stress

**Fig. 4:**
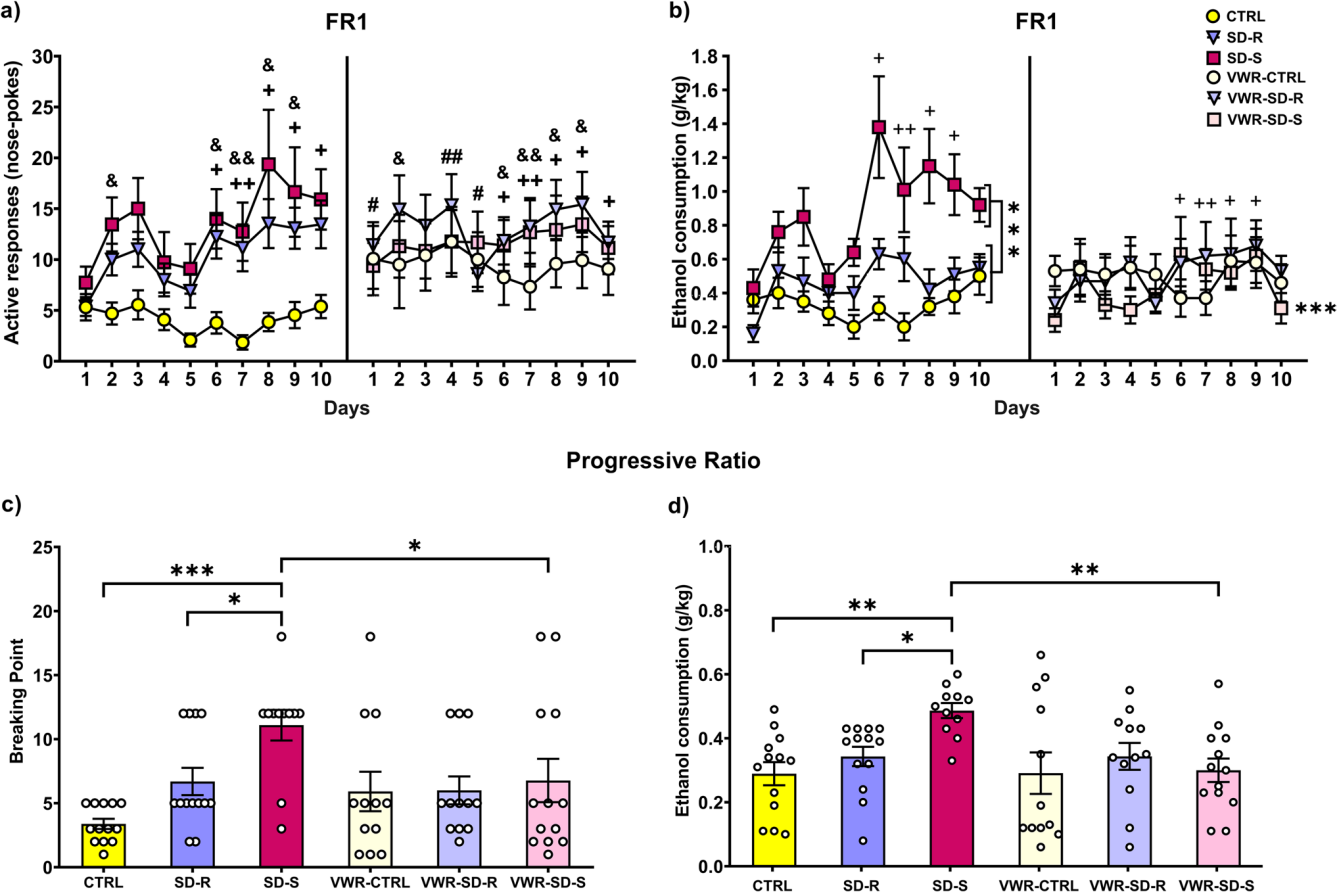
Effects of running wheel on the increase in oral ethanol self-administration induced by social stress in C57BL/6 J mice. The dots represent means and the vertical lines ± SEM of (**a**) the number of active responses and (**b**) the g/kg of ethanol at 20% consumed during FR1 schedule. The columns represent the mean and the vertical lines ± SEM of (**c**) the breaking point values, and (**d**) the g/kg of ethanol at 20% consumed during PR. ***p < 0.001, **p < 0.01, *p < 0.05 significant difference with SD-S group; +  + *p* < 0.01, + *p* < 0.05 significant difference between susceptible mice vs. controls; &&*p* < 0.01, &*p* < 0.05 significant difference between resilient mice vs. controls; ##*p* < 0.01, # *p* < 0.05 significant difference between groups with running wheel access vs. groups without access

No differences were found between the mice during the training phase, showing that SD did not induce any learning deficit (data not shown). The ANOVA for the number of active responses during the FR1 schedule of ethanol SA revealed a significant effect of the interactions Days × SD [F(18,612) = 1.753; *p* < 0.05] and Days × VWR [F(9,630) = 2.158; *p* < 0.05] (Fig. 4a). The post-hoc comparison showed that all mice classified as susceptible (SD-S and VWR-SD-S groups) performed more active responses compared to controls mice (CTRL and VWR-CTRL groups) on Days 6 (*p* < 0.05), 7, 8 (p’s < 0.01), 9 and 10 (p’s < 0.05). Equally, resilient mice (SD-R and VWR-SD-R groups) performed a greater number of effective responses than the control mice (CTRL and VWR-CTRL groups) on Days 2, 6, 7, 8 and 9 (p’s < 0.05). Moreover, the post-hoc comparison showed the performance of a greater number of active responses in VWR-exposed mice compared to sedentary animals on Days 1, 4 and 5 (p’s < 0.05).

With respect to ethanol consumption, the ANOVA revealed a significant effect of the interactions Days × SD [F(18,612) = 2.186; *p* < 0.01) and SD × VWR [F(2,68) = 9.831; *p* < 0.001] (Fig. 4b). The post-hoc comparison showed that the SD-S group consumed significantly more ethanol than the CTRL, SD-R and VWR-SD-S (p’s < 0.001) groups. Additionally, the post-hoc comparison revealed that during Days 9 and 10, resilient mice (SD-R and VWR-SD-R groups) consumed a significantly higher amount of ethanol compared to Day 1 (*p* < 0.05 and *p* < 0.01, respectively).

Also, all mice classified as susceptible (SD-S and VWR-SD-S groups) showed a significantly greater consumption on Days 6 (*p* < 0.05), 8, 9 (p’s < 0.001) and 10 (*p* < 0.01) compared to Day 1, and on Days 6 (*p* < 0.05), 8 (*p* < 0.01) and 9 (*p* < 0.05) compared to Day 4.

During the PR, the ANOVA for the breaking point values and ethanol consumption revealed a significant effect of the interaction SD × VWR [F(2,68) = 3.823; *p* < 0.05] (Fig. 4c) and SD × VWR [F(2,68) = 3.455; *p* < 0.05] (Fig. 4d). The post-hoc comparisons showed that the SD-S group consumed significantly higher amounts of ethanol and achieved a significantly higher BP compared to the CTRL (*p* < 0.01; *p* < 0.001, respectively), SD-R (p’s < 0.05) and VWR-SD-S groups (*p* < 0.01; *p* < 0.05, respectively).

### Exposure to VWR induces an increase in the percentage of mice resilient to increased ethanol intake induced by social defeat

**Fig. 5:**
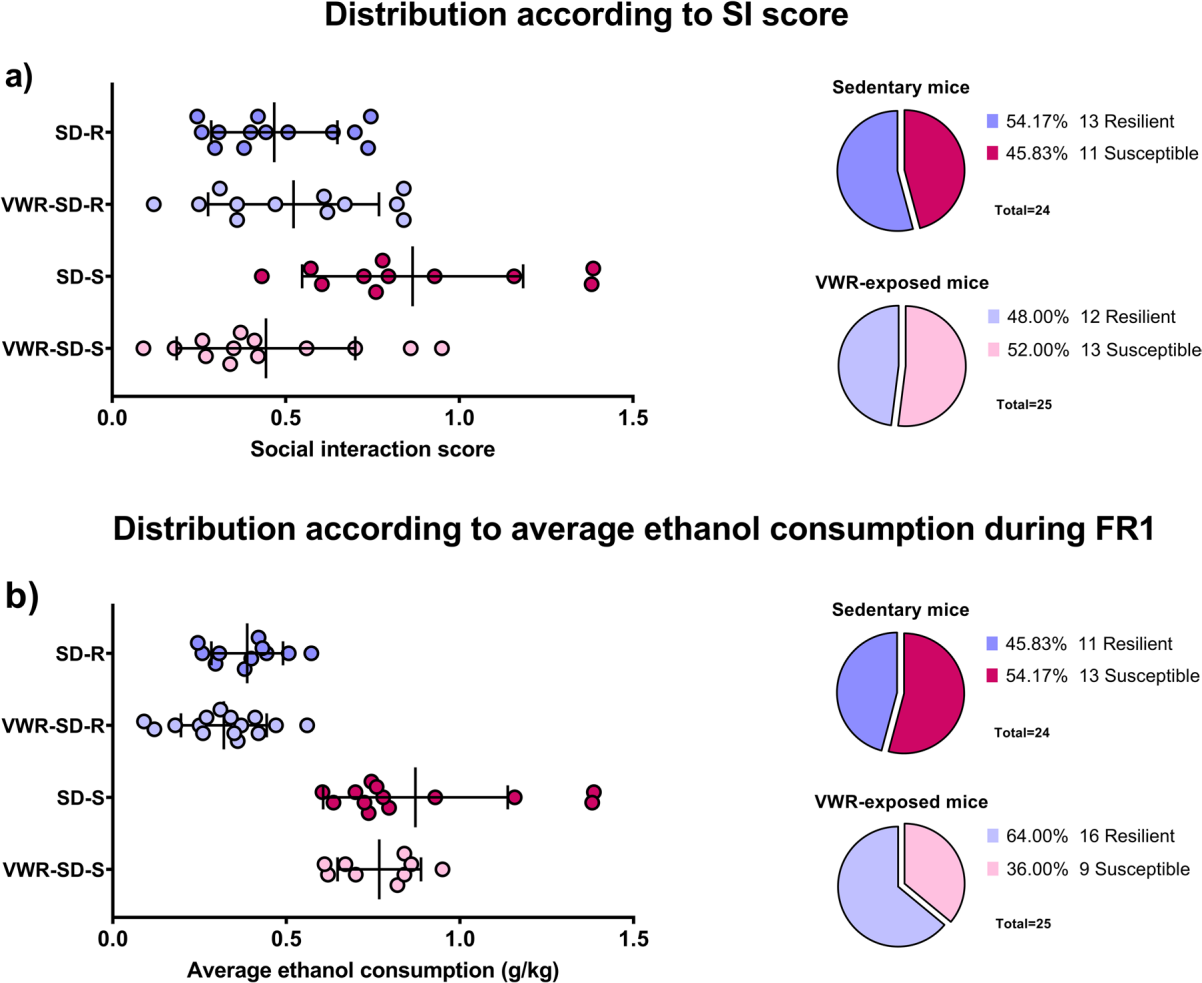
Comparison of the distributions and percentages of subpopulations **a**) according to SIT scores and **b**) according to ethanol consumption during the FR1 schedule of the SA

A K-means cluster analysis was done to see if physical exercise had any effect on mice depending on the alcohol consumption during the FR1 schedule. Sedentary and VWR-exposed mice were classified as Resilient or Susceptible using a cluster analysis [F(1,47) = 95.766; *p* < 0.001] (Fig. 5b).

Figure 5 depicts the distribution diagrams and percentages of susceptible and resilient mice in the sedentary condition and exposure to VWR, as determined by the scores obtained from the SIT (Fig. 5a) and the cluster analysis, which takes into account the average ethanol intake during the FR1 schedule of the SA (Fig. 5b). In both conditions, a change in the distribution of the subpopulations can be observed. We observed that 45.83% of sedentary mice showed a resilient phenotype after ethanol exposure compared to 54.17% observed according to the SIT distribution. In contrast, 64% of VWR-exposed mice showed a resilient phenotype after ethanol exposure compared to 48% observed according to the SIT distribution. Individual changes in phenotype after ethanol exposure are shown in Table 2.

**Table 2:** Individual changes in phenotypes after ethanol exposure

Sedentary mice			VWR-exposed mice		
ID mice	According to SIT Score	According to FR1 intake	ID mice	According to SIT Score	According to FR1 intake
4.1	S	S	13.1	R	R
4.3	S	S	13.3	R	R
4.4	R	R	13.4	S	R
4.5	S	S	13.5	S	R
5.1	S	R	14.1	S	S
5.2	R	S	14.2	S	S
5.3	R	R	14.3	S	S
5.4	R	R	14.4	S	R
6.1	R	R	14.5	R	R
6.2	R	R	15.1	S	R
6.3	S	R	15.2	R	S
6.4	S	S	15.3	R	R
6.5	S	S	15.4	R	S
7.1	R	R	15.5	S	R
7.2	R	S	16.1	S	R
7.3	R	R	16.2	R	S
7.5	S	S	16.3	S	R
8.1	R	S	16.4	R	R
8.2	R	R	17.2	R	S
8.4	R	R	17.4	R	R
8.5	S	S	17.5	S	R
9.1	S	S	18.1	R	S
9.2	R	S	18.2	S	R
9.4	S	S	18.3	S	R
			18.5	R	S

### Social defeat and ethanol exposure induce a decrease in BDNF levels in all susceptible mice

**Fig. 6:**
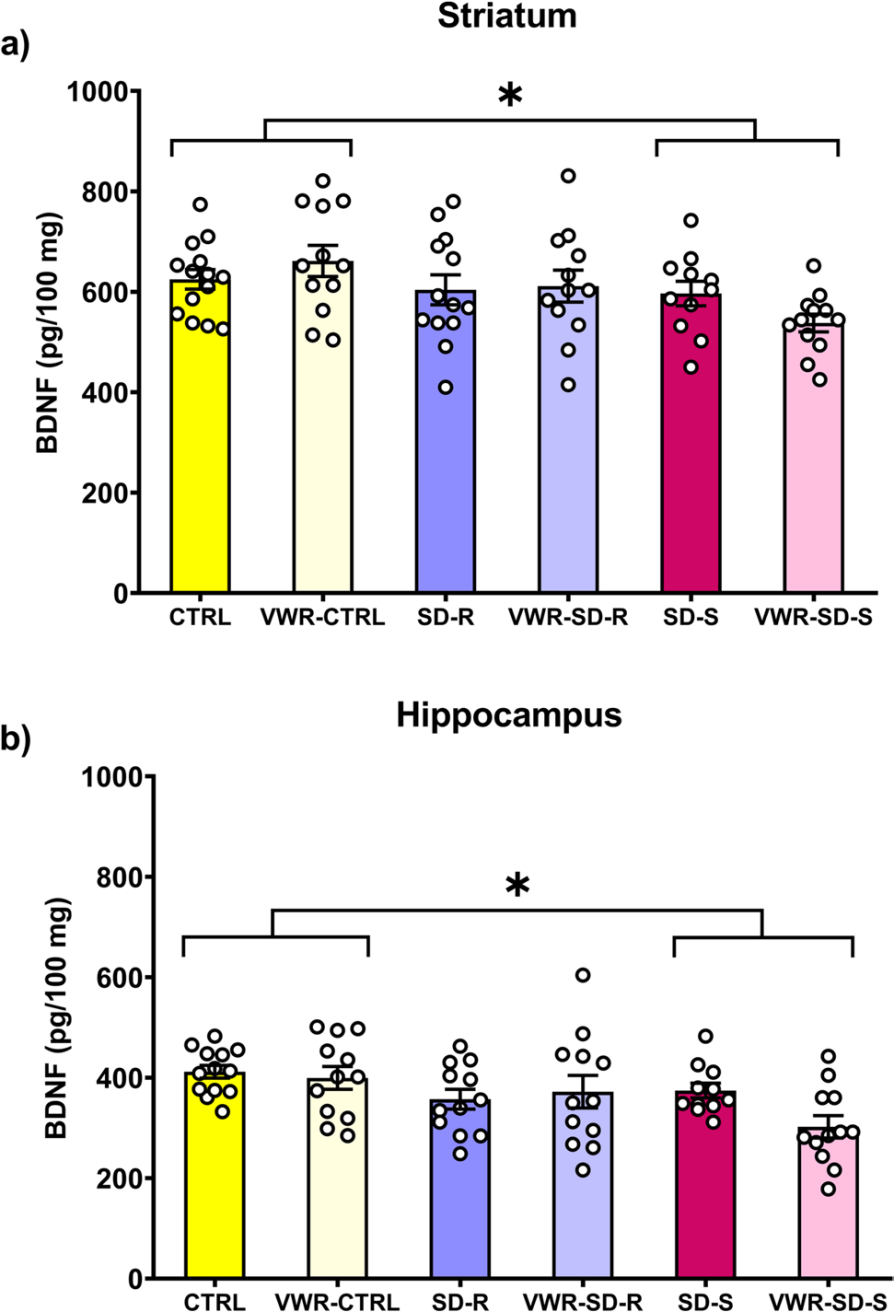
Effects of long-term social defeat on BDNF protein levels in C57BL/6 J mice. The columns represent the mean and the vertical lines ± SEM of the BDNF protein levels (pg/100 mg of protein) in (**a**) the striatum and (**b**) the hippocampus. * *p* < 0.05 significant difference between susceptible mice (SD-S and VWR-SD-S groups) vs. controls mice (CTRL and VWR-CTRL groups)

The ANOVA revealed a significant effect of the variable SD on BDNF protein levels after oral SA of ethanol in the striatum [F (2,68) = 4.125; *p* < 0.05] (Fig. 6a) and the hippocampus [F (2,66) = 4.904; *p* < 0.05] (Fig. 6b). The post-hoc comparison revealed that a significant decrease in BDNF protein levels was observed in all susceptible mice (SD-S and VWR-SD-S groups) compared to controls (CTRL and VWR-CTRL groups) in both areas (p’s < 0.05).

### Low striatal BDNF levels correlated with higher SIT ratio and higher ethanol consumption during FR1 schedule in sedentary resilient mice

**Fig. 7:**
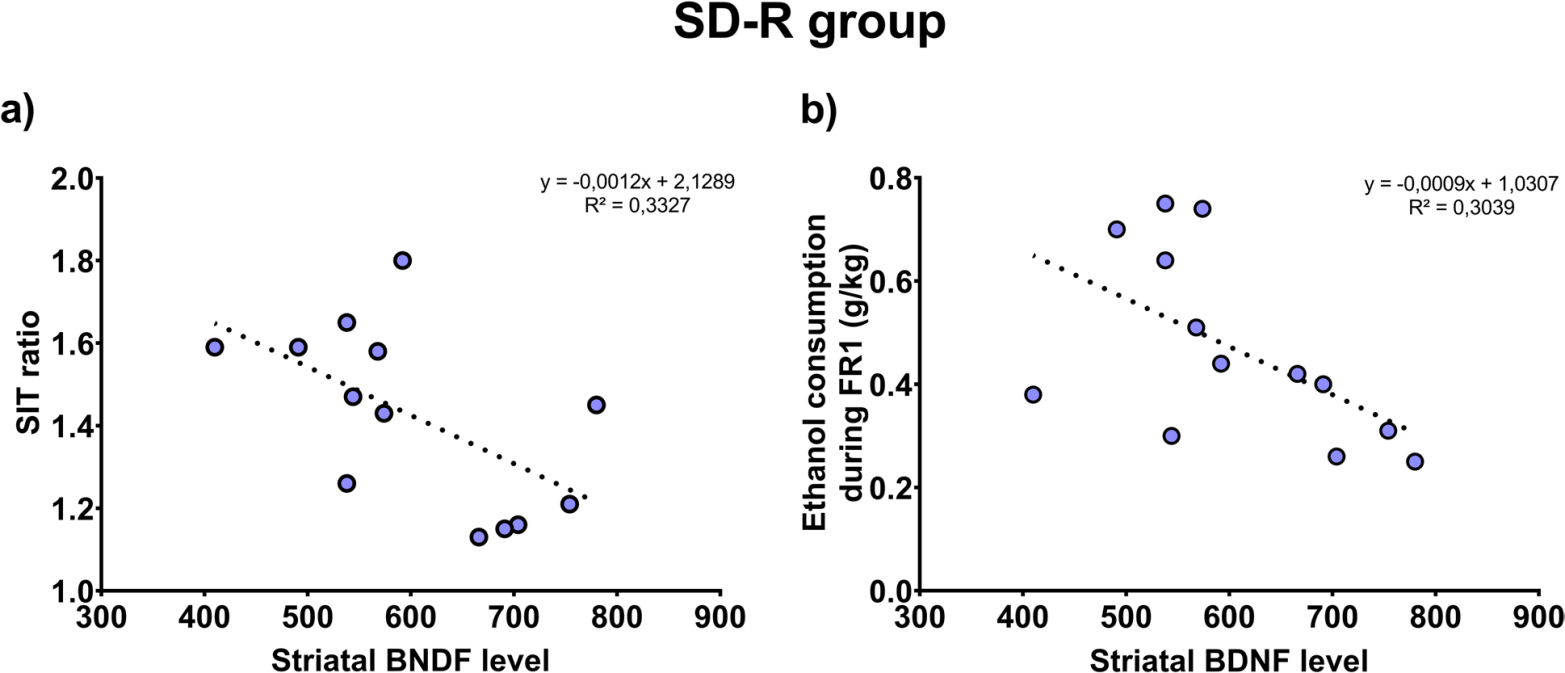
Regression plot for the Pearson correlation between striatal BDNF level and SIT ratio and between striatal BDNF level and average consumption of ethanol during FR1 schedule

Pearson’s coefficient showed a negative correlation between social interaction ratio and the striatal BDNF levels (*r* = -0,577; *p* = 0.039) in the SD-R group (Fig. 7a). Moreover, in this group, we also obtained a negative correlation between the average consumption of ethanol during FR1 schedule and the striatal BDNF levels (*r* = -0.554; *p* = 0.049; Fig. 7b).

## Discussion

Resilience is a complex phenomenon that can be developed and enhanced with various strategies such as the promotion of physical activity (Belcher et al. 2021; McEwen 2006; Southwick et al. 2005). The most important result of the present study is that voluntary physical exercise during adolescence prior to the exposure of SD potentiates a resilient response to ethanol consumption. Physical exercise during adolescence was able to prevent the long-term increase in ethanol consumption induced by social stress in susceptible mice. However, there was no increase in the percentage of mice resilient to depressive-like behavior. In addition, no direct effect of physical exercise on striatal and hippocampal BDNF levels was obtained, with a decrease in protein concentration in all susceptible mice.

### Voluntary wheel running influence the development of the resilient phenotype to depressive-like behaviors

The SIT is an etiologically validated and widely used model for classifying rodents into resilient or susceptible phenotypes based on the appearance of depressive-like behaviors (social avoidance) (Berton et al. 2006; Cathomas et al. 2019; Golden et al. 2011; Krishnan et al. 2007). We observed some differences in the resident and intruder behavior depending on the resilient or susceptible phenotype of the intruder mice during the social defeat encounter, but these were very small. Resilient or susceptible mice are equally at risk of attack from resident mice, with only a slight decrease in time to threat resilient mice exposed to VWR in the last SD. On the other hand, mice that were susceptible to VWR exhibited a decreased duration of defense/submission behaviors during the 1^st^ SD. However, after the experience of several SD, in the 4^th^ encounter, physically exercised, resilient mice spent less time in these behaviors, which suggests enhanced active coping to social stress. On the other hand, exposure to physical activity during adolescence and prior to experiencing SD did not affect the percentage of resilient mice according to the SIT. We reported 46% of resilient mice after exposure to the running wheel versus 54% of sedentary resilient mice. Similarly to the present results, we have previously reported that environmental enrichment applied before SD did not change the percentage of resilient mice to depressive-like behavior measured by the SIT (Reguilón et al. 2021b). Despite the lack of consensus, multiple studies sustain that physical exercise is beneficial in mental disorders related to social stress. It is known that when rodents are exposed to physical exercise during and/or after exposure to SD, social avoidance induced by SD are reversed and a decreased neuroinflammatory response is observed (Ferrer-Pérez et al. 2022; Pagliusi et al. 2020; Reguilón et al. 2020). Nevertheless, only a couple of studies have tested the effect of VWR before SD. In the study of Calpe-López et al. (2022), physical exercise increased resilience to the development of anxiety and apathy/anhedonia assessed by measuring spontaneous grooming behavior with the splash test. The study of Pagliusi et al. (2020) effectively observed that WVR prevents the decrease in the SIT; however, in this study WVR was applied not only before, but during and after the exposure to SD. We have to take into consideration that access to the running wheel prior to a forced swim stress reduced anxious behaviors, but did not affect the endocrine response, with similar increases in corticosterone levels after stress (Lynch et al. 2019).

### Physical activity reduces ethanol intake

The study by Lespine and Tirelli (2018), where three weeks of wheel running exercise during adolescence attenuated the onset and expression of cocaine sensitization in adult mice, led us to hypothesize that adolescence is a particularly sensitive period that may promote long-term resistance to the development of vulnerability to substance abuse use through voluntary physical activity. It is well established that SD produces long-term changes on reward pathways, increasing ethanol preference and consumption (Miczek et al. 2015; Pautassi et al. 2010; Reguilón et al. 2020, 2021a, b; Rodríguez-Arias et al. 2016). In this line, we have previously reported that defeated mice previously housed under environmental enrichment conditions during adolescence did not show an increase in ethanol SA or an increase in immune response.

In the present study, firstly, we have corroborated that SD increased ethanol consumption in susceptible mice. Susceptible mice showed significantly higher ethanol intake than resilient and control mice during the FR1 schedule of oral ethanol SA. Furthermore, during the PR, susceptible mice consumed more ethanol and obtained a higher BP than control and resilient mice to depressive-like behaviors, indicating a higher motivation to obtain the substance. Secondly, although exposure to VWR during adolescence did not increase the resilient response to social avoidance, it proved to be a successful intervention to potentiate resilience to the increased intake and motivation for ethanol. Differently from sedentary mice, there were no differences among control or defeated animals exposed to VWR. Moreover, susceptible mice exposed to VWR showed significantly lower ethanol intake than the sedentary susceptible group during the FR1 schedule of the oral ethanol SA. In addition, susceptible mice exercise with VWR showed lower motivation and ethanol intake during the PR compared to the susceptible group that did not perform physical exercise.

There are several studies that confirmed the protective role of physical exercise on ethanol intake. In a recent report, we reported that VWR during and after the period of SD encounters decreased ethanol consumption and the neuroinflammatory response when compared to defeated mice without access to physical exercise (Reguilón et al. 2020). Using the TBC paradigm, several studies have observed lower consumption in rodents of both sexes with access to physical exercise compared to sedentary animals (Booher et al. 2019; Darlington et al. 2014, 2016; Ehringer et al. 2009; Gallego et al. 2015; Hammer et al. 2010; Piza-Palma et al. 2014). Adolescent mice subjected to physical exercise for two weeks and then exposed to an alcohol binge model showed a reduction in behavioral sensitivity to ethanol intoxication and neuroprotection against ethanol-induced cell death in granule cells in the dentate gyrus of the hippocampus (Leasure and Nixon 2010).

As with drugs of abuse, VWR has rewarding properties, altering gene transcription in the mesolimbic reward pathway and modifying neurotransmitter responses to substance abuse and social stress (Darlington et al. 2014; Greenwood et al. 2011; Herrera et al. 2016; Werme et al. 2002). Acute physical exercise influences the activity of monoamines such as serotonin, dopamine and norepinephrine (Gomez-Merino et al. 2001; Kobayashi et al. 2022; Lin and Kuo 2013), and long-term exercise induces adaptations in serotonergic 1A receptors in the raphe nuclei and dopamine receptor D2 in the striatum (Bauer et al. 2020; Clark et al. 2015; Greenwood 2019). Therefore, physical exercise-induced adaptations in monoamines may alter their response during ethanol intake and lead to a reduced vulnerability to develop problematic alcohol consumption (Buhr et al. 2021).

Taking all these data into account, we wanted to examine whether physical exercise had any effect on ethanol intake by re-classifying mice into susceptible and resilient subpopulations according to average ethanol intake during FR1 of SA. We observed changes in the distribution of the subpopulations. Following the new distribution, we detected that 45.83% of sedentary mice showed a resilient phenotype after ethanol exposure compared to 54.17% observed according to the SIT distribution. In contrast, 64% of VWR-exposed mice showed a resilient phenotype after ethanol exposure, compared to 48% observed according to the SIT distribution. Therefore, considering these data, we can indicate that exposure to physical exercise during adolescence induced an increase in the resilience phenotype to social stress-induced increased alcohol intake in adulthood.

### Decrease in striatal and hippocampal BDNF levels in susceptible mice

The neurotrophin BDNF is widely distributed in the central and peripheral nervous system. The control of survival, growth and differentiation of specific neuronal populations are among the major functions of BDNF (Barde et al. 1987; Park and Poo 2013; Zagrebelsky and Korte 2014). In addition, BDNF has been widely implicated in the development of mood and addictive disorders (Bandelow et al. 2017; Feltenstein and See 2013; Lüscher and Malenka 2011; Nestler 2013; Nikulina et al. 2014). SD in rodents results in increased BDNF expression in several brain areas such as the PFC (Ferrer-Pérez et al. 2019), amygdala, and the bed nucleus of the stria terminalis (Vasconcelos et al. 2021), NAc and VTA (Miczek et al. 2011; Fanous et al. 2010; Berton et al. 2006; Krishnan et al. 2007). Bergström et al. (2008) reported an upregulation of BDNF mRNA in the ventral hippocampus of resilient rats after exposure to chronic mild stress. Therefore, we must point out that susceptible animals show response mechanisms that differently affect BDNF signaling.

In this study, we observed that BDNF levels in the striatum correlate negatively with SIT scores. This suggests that as BDNF levels in the striatum increase, there is a decrease in social interaction skills of resilient mice, a result that has been observed in other studies that have evaluated the mesolimbic system (Krishnan et al. 2007). It is important to note that resilient mice did not show social avoidance with a SIT ratio equal to or higher than 1. On the other hand, it is known that the performance of physical exercise on VWR produces transcriptional and post-transcriptional regulation of BDNF in different brain areas (Venezia et al. 2016; Cotman and Engesser-Cesar 2002). In the hippocampus, increases in BDNF expression have been observed in adult and adolescent male and female rodents after 1 and up to 28 weeks of training (Gallego et al. 2015; Griesbach et al. 2004; Lee and Soya 2017; Seifert et al. 2010; Venezia et al. 2016). Increases in the frontal cortex have also been observed after three weeks of physical exercise in adult male rats (Graban et al. 2017). Equally, we have reported that exposure to VWR during SD can induce an increase in BDNF levels in the striatum and hippocampus (Ferrer-Pérez et al. 2022).

However, there are no studies evaluating the changes in BDNF in defeated rodents previously exposed to running wheels. Some studies using physical stress (restraint) showed that three to six weeks of voluntary physical activity prior to stress counteracts the decrease in BDNF expression in the hippocampus (Adlard and Cotman 2004; Greenwood et al. 2007; Lapmanee et al. 2017). In the present study, we did not observe any long-term effect of VWR on BDNF level measured after wheel removal in control or resilient mice. This lack of effect could be due to the experimental procedure, since there was a period of nine weeks between the last session of exercise and the analysis of BDNF levels. Increases in BDNF levels induced by physical activity usually decrease after 14 days of wheel removal (Berchtold et al. 2010).

It is also important to consider the effects of ethanol intake. Acute administration induces increases in BDNF in the hippocampus, but decreases have been observed after chronic exposure (Logrip et al. 2015; Silva-Peña et al. 2019; Yang et al. 2017). When alcohol consumption is chronic, a dysregulation of BDNF expression in the dorsal striatum occurs, thereby promoting an increase in alcohol intake (Logrip et al. 2009). Also, it has been observed that prolonged intake of high volumes of ethanol (20%) significantly reduce BDNF expression in PFCm (Darcq et al. 2015).

Regarding the effect of ethanol on BDNF function in combination with physical exercise, has been little studied, discrepancies have been observed. On the one hand, 21 days of VWR during ethanol exposure by TBC induced an increase in BDNF expression in the hippocampus (Gallego et al. 2015). On the other hand, in another study, a decrease in BDNF expression in the hippocampus was observed after 16 days of physical exercise during ethanol exposure compared to mice in the physical exercise condition without ethanol exposure (Darlington et al. 2014).

In the present study, a decrease in the levels of BDNF protein was observed in both the striatum and hippocampus of susceptible mice, with and without access to running wheels, in comparison to the control groups (CTRL and CTRL-VWR). Although these results do not correlate with those previously observed in the scientific literature in striatal areas (Berton et al. 2006; Krishnan et al. 2007; Miczek et al. 2011), numerous methodological approaches have been used. Considering the period since animals were allowed to exercise, it is not surprising that physical exercise has not had a significant effect on BDNF levels. Given that BDNF levels in the striatum correlate negatively with ethanol intake during the FR1 schedule of SA, we hypothesized that prolonged high-concentration alcohol consumption (20%) could interfere with this adaptive mechanism by pronouncedly decreasing BDNF levels in the striatum of susceptible mice.

## Conclusions

To sum up, the resilient phenotype to SD develops at different levels, such as depressive-like behaviors, ethanol consumption and BDNF changes. Our results point to the protective role of VWR in potentiating resilience to some SD effects on ethanol intake. Despite the lack of consensus, our results strongly suggest that BDNF signaling in susceptible mice is differentially affected by ethanol intake compared to resilient animals. Voluntary physical exercise during adolescence is a beneficial environmental intervention with long-term power to promote resilience to the increased vulnerability to ethanol intake induced by social defeat. Encouraging healthy physical activity habits seems to be beneficial for an adaptive response to social stress and to protect against the development of problematic alcohol consumption.

## Supplementary Information

Below is the link to the electronic supplementary material.

Supplementary file1 (MP4 949 KB)

Supplementary file2 (MP4 1819 KB)

Supplementary file3 (MP4 1756 KB)

Supplementary file4 (MP4 851 KB)

Supplementary file5 (DOCX 59 KB)

Supplementary file6 (PNG 179 kb)

High resolution image (TIF 2899 kb)

Supplementary file7 (XLSX 30 KB)

## Data Availability

The data that support the findings of this study are available on request from the corresponding author [M.R.A].

## Abbreviations

BDNF: Brain-derived neurotrophic factor
BP: Breaking point
CPP: Conditioned place preference
DID: Drinking in the dark
ELISA: Enzyme-linked immunosorbent assay
FR1: Fixed ratio 1
HPA: Hypothalamic-pituitary-adrenal
NAc: Nucleus accumbens
PND: Postnatal day
PR: Progressive ratio
SA: Self-administration
SD: Social defeat
SIT: Social interaction test
TBC: Two-bottle choice
VTA: Ventral tegmental area
VWR: Voluntary wheel running
