# Supplementary material for: Voluntary wheel running during adolescence prevents the increase in ethanol intake induced by social defeat in male mice
Source: Psychopharmacology (Berl). 2023 Sep 22;242(5):979–96. doi: 10.1007/s00213-023-06461-0 (PMC12043745; doi:10.1007/s00213-023-06461-0)
Supplement: Supplementary file 5 — Supplementary file5 (DOCX 59 KB) [file 213_2023_6461_MOESM5_ESM.docx]

**Supplementary Information for the Article:**

Voluntary wheel running during adolescence prevents the increase in ethanol intake induced by social defeat in male mice

**This material supplements, but does not replace, the peer-reviewed paper in Psychopharmacology.**

Marina D Reguilón^1^, Carmen Ferrer-Pérez^2^, Carmen Manzanedo^1^, José Miñarro^1^, Marta Rodríguez-Arias^1^

^1^Unidad de Investigación Psicobiología de las Drogodependencias, Departamento de Psicobiología, Facultad de Psicología, Universitat de València, Valencia, Spain.

^2^Departmento de Psicología Evolutiva, Facultad de Psicología, Universitat de València, Valencia, Spain

**Correspondence author:**

Dr. Marta Rodríguez-Arias. Department of Psychobiology, Facultad de Psicología, Universitat de Valencia, Avda. Blasco Ibáñez, 21, 46010 Valencia, Spain Tel.: +34 - 96 386 46 37; Fax: +34 - 96 386 46 68; e-mail: [marta.rodriguez@uv.es](mailto:marta.rodriguez@uv.es)

**Results**

**1. Body weight during the weeks of ethanol access in the defeated groups.**

The ANOVA of the body weight during the period of ethanol access did not reveal any significant differences between- and within-subject variables. Therefore, no significant differences in body weight were observed among the experimental groups (Fig. A).

**Figure A. Body weights during the DID and SA weeks induced by social stress in C57BL/6J mice.** The dots represent means and the vertical lines ± SEM of the body weights.
