# Supplementary figures and images for: Voluntary wheel running during adolescence prevents the increase in ethanol intake induced by social defeat in male mice
Source: Psychopharmacology (Berl). 2023 Sep 22;242(5):979–96. doi: 10.1007/s00213-023-06461-0 (PMC12043745; doi:10.1007/s00213-023-06461-0)

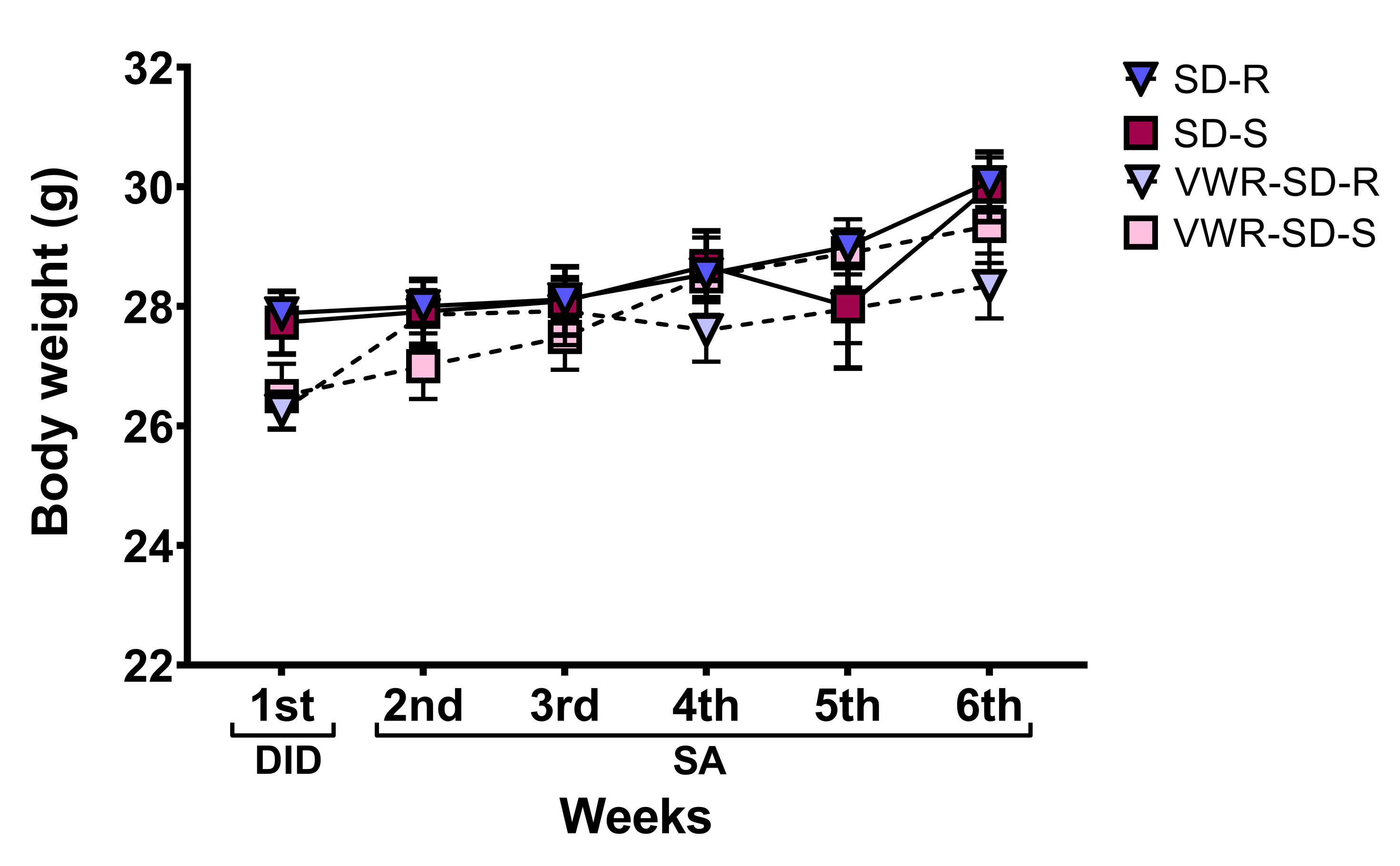

Supplement: Supplementary file 6 — Supplementary file6 (PNG 179 kb) [file 213_2023_6461_Fig8_ESM.png]
